# Self-management experience in adults with colostomies due to colorectal cancer

**DOI:** 10.15649/cuidarte.4265

**Published:** 2025-07-07

**Authors:** Lina Marcela Zuluaga Sarmiento, Gloria Mabel Carrillo González

**Affiliations:** 1 Nurse, Oncology Nursing Specialist; Master’s in Nursing. Faculty of Nursing, Universidad Nacional de Colombia. Bogotá, Colombia. E-mail: lzuluagas@unal.edu.co Universidad Nacional de Colombia Bogotá Colombia lzuluagas@unal.edu.co; 2 Nurse; Master’s in Nursing; PhD in Nursing; Full Professor. Faculty of Nursing, Universidad Nacional de Colombia. E-mail: gmcarrillog@unal.edu.co Universidad Nacional de Colombia Bogotá Colombia gmcarrillog@unal.edu.co

**Keywords:** Self-Management, Life Experiences, Colorectal Cancer, Adult, Colostomy, Automanejo, Experiencias de Vida, Cáncer Colorrectal, Adulto, Colostomía, Autogestão, Experiências de Vida, Câncer Colorretal, Adulto, Colostomia

## Abstract

**Introduction:**

Facing a chronic disease such as colorectal cancer with a colostomy is a process that represents changes in people's quality of life. Addressing this experience is an enriching process that strengthens self-management interventions.

**Objective:**

To describe the self-management experience of adults with colostomy due to colorectal cancer.

**Materials and Methods:**

A qualitative study with a descriptive phenomenological approach was conducted. Ten people over 18 years of age with colorectal cancer with temporary or permanent colostomies of at least 6 months' evolution participated voluntarily in semi-structured interviews. Data were analyzed using the Colaizzi analysis method.

**Results:**

Eight categories are associated with the phenomenon, and thirty nominal codes represent the experience. The spiritual dimension, social support, coping with colostomy-related difficulties, and experiencing psychosocial changes were identified as factors that influence self-management, as well as practices and behaviors, such as self-management skills, living a new reality, adapting to the colostomy, and support and learning from the healthcare team and system.

**Discussion:**

The difficulties experienced, family and social support, social effects, and spiritual support are consistent with the literature. This research highlights the difficulties with access to supplies, helping others, nicknaming the stoma, and the interactions with the health system.

**Conclusion:**

Knowing the self-management experience, influencing factors, and practices contributes to implementing interventions with a better impact aimed at improving the quality of life and the new life experience of having a colostomy.

## Resumen

**Introducción:**

Enfrentar una enfermedad crónica como el cáncer colorrectal con colostomías es un proceso que representa modificaciones en la calidad de vida de las personas. El abordaje de la experiencia es un proceso enriquecedor para fortalecer las intervenciones en automanejo.

**Objetivo:**

Describir la experiencia de automanejo en adultos con colostomía por cáncer colorrectal.

**Materiales y métodos:**

Estudio de enfoque cualitativo fenomenológico descriptivo, 10 personas mayores de 18 años con cáncer colorrectal con colostomías temporales o permanentes de mínimo 6 meses de evolución, que aceptaron participar voluntariamente en las entrevistas semiestructuradas, acogiendo el método de análisis de Colaizzi.

**Resultados:**

Ocho categorías asociadas al fenómeno y treinta códigos nominales representan la experiencia. Se identificaron la dimensión espiritual, el apoyo social, afrontar dificultades con la colostomía y vivir cambios psicosociales como factores que influyen en el automanejo, así como las prácticas y conductas, tales como las habilidades de automanejo, vivir una nueva realidad, adaptándose a la colostomía y el apoyo y aprendizaje desde el equipo y sistema de salud.

**Discusión:**

Las dificultades experimentadas, el apoyo familiar y social, los efectos sociales y el apoyo espiritual son consistentes con la literatura. De esta investigación se resalta las dificultades con el acceso a insumos, la ayuda a otros, las denominaciones y la relación con el sistema de salud.

**Conclusiones:**

El conocimiento de la experiencia en automanejo, los factores y las prácticas aportan en la realización de intervenciones con mejor impacto orientadas a mejorar la calidad de vida y la nueva vivencia de tener una colostomía.

## Resumo

**Introdução:**

Enfrentar uma doença crônica como o câncer colorretal com colostomias é um processo que representa modificações na qualidade de vida das pessoas. Abordar a experiência é um processo enriquecedor para fortalecer intervenções de autogestão.

**Objetivo:**

Descrever a experiência de autogestão em adultos com colostomia devido ao câncer colorretal.

**Materiais e métodos:**

Estudo qualitativo fenomenológico descritivo com 10 pessoas maiores de 18 anos, portadoras de câncer colorretal e colostomias temporárias ou definitivas com duração mínima de 6 meses, que voluntariamente aceitaram participar de entrevistas semiestruturadas, utilizando o método de análise de Colaizzi.

**Resultados:**

Oito categorias associadas ao fenômeno e trinta códigos nominais representam a experiência. A dimensão espiritual, o apoio social, o enfrentamento das dificuldades com a colostomia e a vivência de mudanças psicossociais foram identificados como fatores que influenciam o autogerenciamento, assim como práticas e comportamentos como habilidades de autogerenciamento, viver uma nova realidade, adaptar-se à colostomia e apoio e aprendizado da equipe e do sistema de saúde.

**Discussão:**

As dificuldades vivenciadas, o apoio familiar e social, os efeitos sociais e o apoio espiritual são consistentes com a literatura. Esta pesquisa destaca as dificuldades de acesso a suprimentos, ajuda ao próximo, denominações e relacionamento com o sistema de saúde.

**Conclusões:**

O conhecimento da experiência, dos fatores e das práticas de autogestão contribui para a implementação de intervenções de maior impacto, visando à melhoria da qualidade de vida e da nova experiência de ter uma colostomia.

## Introduction

Facing a chronic disease such as cancer is a process that involves a series of changes in the lifestyles and quality of life of both patients and their families. Ongoing efforts to reduce cancer-related morbidity and mortality rates continue to advance^1^-^3^, aiming to minimize its family and social impact through regulations, public policies, and care strategies^4^-^6^ that promote the best and greater involvement of individuals in their own care process, such as self-management. 

In Colombia, colorectal cancer is the third leading cause of death for both sexes and has shown a steady increase in incidence in recent years^7^-^10^. It imposes a substantial personal, familial, and societal burden of the disease throughout both treatment and survivorship^11^-^14^. Developing self-management behaviors fosters collaboration between adults with colorectal cancer and healthcare teams to define priorities according to the problems encountered, set goals, create action plans, and develop problem-solving skills^15^. One of the most enriching processes to strengthen self-management interventions is exploring the experiences of individuals living with chronic conditions to understand possible situations that may hinder their self-management^16^-^20^. 

Life for people with colorectal cancer changes dramatically, disarticulating their daily life routines as well as their bodily and corporality experience^21^. Daily life changes, making it impossible to do usual activities, and the change is even more drastic when a colostomy is required, often accompanied by uncertainty and increased burden. Individuals with colorectal cancer who undergo a colostomy must redefine aspects of their lives, including diet, hygiene, family relationships, and healthcare^21^, since healthy behavior is complex and requires the integration of self-management practices into the lifestyles of both individuals and their families^22^. 

Knowing how the self-management experience is among adults with colostomy due to colorectal cancer, with a knowledge construction view, allows the design of interventions to support follow-up care for people with this condition. Qualitative research makes it possible to study human experience, feelings, and emotions, as well as the objective and subjective characteristics of care, as part of the self-management intervention process, recognizing the phenomenon as it unfolds, giving a unique and inestimable value to holistic nursing care beyond the biological dimension. 

The aim of this research was to describe the self-management experiences of adults with colostomy due to colorectal cancer. 

## Materials and Methods

**Design**

This study used a qualitative approach with a Husserlian descriptive phenomenological design. This design aimed to provide a description of the phenomenon under study, supporting critical and reflective analysis to enhance nursing care. 

**Participants**

The study population consisted of adults with colostomies due to colorectal cancer in Bogotá. The participants met the following inclusion criteria: individuals over 18 years of age who voluntarily agreed to participate in the study; diagnosed with colon or rectal cancer at any stage, either undergoing active treatment or in survivorship; with temporary or permanent colostomies for at least six months; and enrolled in a wound care or ostomy program. 

Exclusion criteria included individuals with mild cognitive impairment, dementia, or communication and language difficulties. Also excluded were individuals whose colostomies were performed as part of bowel diversion treatments for other oncological diagnoses. 

The sample size was determined based on theoretical saturation, according to the quantity and quality of the information, to the point of not obtaining new insights that could further contribute to or deepen the understanding of the phenomenon. 

**Data collection**

Data were collected through observation and semi-structured interviews, which were recorded in a field log and audio recorded. Interviews were transcribed immediately after completion and managed using ATLAS.ti software. 

Examples of guiding questions included:

• Describe what the experience of living with this colostomy has been like for you, starting from when it was first placed.

• Try to recall how you felt at each moment of the situation.

• What has been your experience with self-management of your health condition? Please, try to recall and comment on any positive and/or negative situations related to self-management.

On average, each interview lasted 53 minutes, for a total of 538 minutes. Interviews were coded using the following structure: E (Interview), followed by the interview number (1, 2, 3, etc.); P (Participant), followed by the participant number (1, 2, 3, etc.); and M or F to indicate the participant's sex (Male or Female), for example: E1P1M, E2P2F. 

The study adhered to methodological rigor criteria based on dependability, credibility, transferability, and confidentiality. 

Resolution 8430 of 1993^23^ was taken into account, which outlines the scientific, technical, and administrative standards for health research in Colombia, including the requirement for informed consent. It also followed the International Ethical Guidelines for Biomedical Research Involving Human Subjects issued by the Council for International Organizations of Medical Sciences (CIOMS), in collaboration with the World Health Organization (WHO)^24^. 

The study was approved by the Ethics Committee of the Faculty of Nursing at the Universidad Nacional de Colombia and the Convatec Research Committee. 

**Analysis framework**

Colaizzi's method was used for the analysis process^25^, which involves seven steps: familiarization through reading and rereading transcripts, extraction of significant statements, formulation of meanings, clustering of themes, development of an exhaustive description of the phenomenon, identification of the fundamental structure, and validation of this structure by returning to the participants for confirmation. The collected data are available for free access and consultation in Mendeley Data^26^. 

## Results

Ten participants who met the inclusion criteria were enrolled. Among them, two were women, and eight were men, ranging in age from 20 to 90 years, with an average age of 65 years. The data related to age range, marital status, place of origin, socioeconomic status, and occupation are detailed in Table 1. 

**Table 1:** Sociodemographic characteristics

Participant	Sex	Age	Medical Diagnosis	Occupation	Marital status	Education Level	Socioeconomic status	Ostomy type	Duration
1	M	68	Colon cancer	Self-employed	Single (with partner)	High school	3	Permanent colostomy	1-2 years
2	M	69	Colon cancer	Retired	Married	High school	3	Temporary colostomy	1-2 years
3	M	74	Colon cancer	Self-employed	Married	High school	3	Temporary colostomy	< 1 year
4	F	55	Colon cancer	Disabled (pension pending)	Married	High school	3	Permanent colostomy	1-2 years
5	M	80	Colon cancer	Unemployed	Widowed	Elementary school	3	Temporary colostomy	1-2 years
6	F	61	Rectum Cancer	Housemaker	Separated	High school	3	Temporary colostomy	> 2 years
7	M	64	Colon cancer	Self-employed	Married	Elementary school	2	Permanent colostomy	> 2 years
8	M	20	Rectum Cancer	Employed	Single	High school	2	Temporary colostomy	< 1 year
9	M	72	Colon cancer	Self-employed	Married	Elementary school	2	Temporary colostomy	1-2 years
10	M	90	Colon cancer	Retired	Widower	Elementary school	5	Permanent colostomy	< 1 year

Source: Adapted from Master's Thesis Zuluaga L, 2023.

**Experience**

A total of 1074 descriptive codes were identified, from which eight categories and 30 nominal codes emerged, all related to the phenomenon of self-management in individuals with colostomies due to colorectal cancer. The identified categories are shown in Table 2. 

Self-management experience of adults with colostomies due to colorectal cancer can be described as an experience that encompasses different factors and characteristics. These include the spiritual dimension, the presence of social support, the difficulties of coping with the colostomy, and the psychosocial changes. It also includes practices and behaviors such as practicing self-management skills and living in a new reality that entails an adaptation process influenced by the support and education from the healthcare system and its team. 

Factors influencing self-management in adults with colostomy due to colorectal cancer can be associated with participants' perceptions and with cultural, social, protective, and economic factors. Cultural factors involve the environments where they live, social relationships, and family characteristics, which help shape behaviors. Social factors include the influence of friends or others and make visible cultural characteristics that shape behavior in response to a new life experience. Economic factors, such as financial changes for access to available supplies for colostomy care, also influence self-management. 

**Table 2:** Categories and nominal codes of self-management experience in adults with colostomies due to colorectal cancer

Category	Nominal Codes
1. Coping with colostomy difficulties	Colostomy-related accidents: leakage Initial difficulties with the colostomy Difficulties controlling gas Difficulties in colostomy management Difficulties in accessing colostomy management supplies Difficulties in accessing health services Difficulties resting and sleeping
2. Living a new reality	Adapting to life with a colostomy Colostomy learning through unfolding events Helping others through experience Controlling physical symptoms Beliefs associated with colostomy
3. Receiving social support	Support from the partner Support from the family General social support Technological support Peer-to-peer support
4. Adapting to the colostomy	Becoming familiar with the colostomy Nicknaming the colostomy Understanding the colostomy - Resolving doubts Feelings associated with having a colostomy
5. Experiencing psychosocial changes	Additional financial costs Adjustments in work roles Social limitations
6. Practicing colostomy self-management skills.	Exploration of homemade items for colostomy management Colostomy self-management practices Dietary self-management practices
7. Learning about self-management with the health care team and system.	Support from healthcare providers Knowing the administrative procedures
8.Strengthening the spiritual dimension	Relationship with a supreme being

Source: Adapted from Master's Thesis Zuluaga L, 2023.

Among the protective factors, spirituality emerged as an important support for all participants in the experience of navigating the difficulties of having a colostomy due to colorectal cancer. Time also functioned as a protective factor, as coping with colostomy varies according to the moment of the experience, and the impact tended to diminish over time. 

Behaviors and practices influencing colostomy self-management among colorectal cancer patients are determined by the development of skills and abilities for colostomy care. They are also determined by new conditions and life perspectives, such as shifts in body image, the adaptation processes involving feelings, and how these also influence the life experience. 

Practices and their modification over time are directly related to the guidance and support of the healthcare system and its professionals, who contribute to colostomy education and care, as well as access to necessary care services and resources. 

Category N° 1: In the context of individuals with colostomies due to colorectal cancer, several challenges were identified, particularly during the initial phase of the process. These included difficulties related to colostomy management and control, leakages, gas control, difficulties in accessing supplies and health services, and disruptions to rest and sleep. Collectively, these issues imply a disruption to daily life but also provide opportunities for experiential learning and the future development of self-management knowledge and decision-making skills. 

Difficult events are determined by the meanings, settings, and moments they occur, each presenting an obstacle to be overcome. The early stages of the process were described as difficult due to the demands of care, discomfort, changes in body image, insufficient explanations, and resistance to accepting the process.

*"One time, I had this habit of taking the bag off to wash it. So, I took it off, and of course, I had loose stools—and I didn't expect it to gush out so badly. After that, I never took it off like that again." E8P8M 8:58 *

*"The beginning was really hard. Really, really hard. Because, well… that thing—it's like a living part there. You have to take care of it, so it doesn't bump into anything. Sometimes, the thing itself… it sort of comes alive, you know? And sometimes, it even bleeds." E1P1M 1:36 *

Category N° 2: The category "living a new reality" emerges from participants’ reflections on the changes they experience as a result of living with a colostomy, as well as the learning that arises from that experience. It is described as a step-by-step process involving both positive and negative aspects, which ultimately leads to a sense of routine and clearer learning that allows individuals to experience life in a way that feels more normal. 

Having a colostomy is perceived as a difficult experience, one to which individuals do not get used to. It is described as "endurable," depending on the knowledge gained and the desire to move forward. The changes mentioned relate to the transformation of living conditions and lifestyle, and the profound impact on their current way of life. However, participants highlight the association between having a colostomy and staying alive, framing the experience as one that ultimately offers hope.

*"It’s tough, really tough… it's hard. It's complicated—complicated in the sense that it changes your life. It really does. Your diet changes a lot too, because… well, managing this is difficult." E7P7M 7:6*

*"People don't even realize I have this, but yeah—I pretty much always have to stay covered. I can’t wear my shirt the way I used to, and my pants... I can't pull them all the way up anymore. I have to wear them lower." E1P1M 1:173*

Category N° 3: Within the experience of living with a colostomy, participants emphasized the importance of relationships and the support they require for care and daily activities. These support systems operate within various contexts. In the case of partners, support may include assistance with physical care, feeding, and emotional well-being. Within the family context, participants highlighted emotional support, a sense of recognition, and the value of maintaining peaceful family dynamics. Technological support is another current aspect of people's daily lives that is very important and supports colostomy care, such as the use of the internet.

 In the experience of having a colostomy and developing self-management strategies, participants' relationships with different support systems are relevant. These relationships have an effect such as influence, support, collaboration, and information.

*"No, no—when I need to talk to someone, I talk to my children, or my brothers, my sister—I have a sister who helps me a lot. And my wife too—she’s the one who takes care of everything around here, and she helps a lot." E2P2M 2:77*

Category No. 4: Having a colostomy involves an adaptive process requiring a series of conditions related to information and education. The education received, from nursing personnel training, signs on when to seek emergency care, instructions for management, and explanations provided at specialized institutions, to the timing of educational sessions and the training for both patients and families, are aspects that had a great influence, both positive and negative, on participants' experience of having a colostomy. 

Adaptation and acceptance included resolving doubts about the colostomy at various process stages, such as the potential for future anastomosis or access to educational appointments.

*"I had chemo and radiation, but they couldn’t shrink the tumor enough, so I ended up having surgery to remove it completely. After that—around December, maybe the 20th or 22nd—I started using the colostomy, and I've had it since then." E2P2M 2:2*

As part of the acceptance process, the participants used different terms to refer to their stoma, such as "little gut," "member," "bow tie of living flesh," which is part of the relationship developed with this different part of their body and the care it requires. Using this language helped make the experience more familiar, clearer, and personalized.

*"With that distress, and I'm holding everything in… that's when it really starts acting up. Nothing to do about it. I call it little gut—it gets upset and just lets everything out." E5P5M 5:29*

Finally, the feelings associated with having a colostomy play an important role in adaptation. They cover a wide range, including both positive and negative emotions, such as despair, shame, contentment, and happiness, among others.

*"Well... no, it doesn't really feel good, because this is just another struggle of human being's life. It is not the same, people like you who can go about things normally. With this, you have to be more careful. But still, not to the point of getting sad about it." E10P10M 10:39*

Category No. 5: “Experiencing psychosocial changes” covers all the components related to economic and occupational issues, as well as the social limitations faced by people living with a colostomy. The additional financial costs of maintaining adequate resources to support the new demands, on top of routine expenses, have significant implications for participants and their families. Budget increases are often necessary to ensure proper care contributing to better physical well-being.

*"They give me several sheets —10 per month, 10 monthly and for 10 bags—so I end up paying about 47,000 pesos. Then I have to go back and ask for these that are supposed to be the generic ones, and I only pay around 3,000." E1P1M 1:116.*

Social limitations, particularly odor-related, were mentioned and associated with changes in moments such as social gatherings with friends or family, traveling, or using public transportation. They require special attention to care routines to prevent unpleasant incidents, which often lead to feelings of rejection or ridicule.

*"No, no, the smell… the smell can be hard sometimes. Like, if you're in a meeting and the bag inflates, you have to excuse yourself, leave the room, go somewhere far, and let the air out of the bag because—there" E1P1M 1:72.*

Category N° 6: Having a colostomy due to a diagnosis of colorectal cancer involves the need to learn practical tools independently, establish a routine, determine the frequency of self-care activities, and explore daily care alternatives. This need is closely linked to the development of instrumental skills for managing and caring for the colostomy. 

Exploring the use of household items, such as belts, sanitary pads, gloves, and aprons, is one homemade strategy reported by the participants for colostomy care, including managing pouches, controlling symptoms, and addressing difficult situations. Notably, the use of these homemade items was largely based on the participants' own decisions and resourcefulness.

*"I came up with this little thing because sometimes the bag comes loose and, oh man, by the time you notice, it’s already leaking. It's like a belt, yeah—and when it fills up with gas, you have to lift this little thing to let the gas out. But you've got to do it far away because —well, you know." E1P1M 1:30*

Changes in diet over time are different and more controlled at the beginning of the experience, gradually evolving into a more "normal" pattern as time passes.

*"Well, look, I am going to be honest. I stuck to the diet for the first two or three months. But now, I just eat whatever we’re having at home—more potatoes, rice, meat, fruits, vegetables… whatever I can find. One thing's for sure, I haven't lost my appetite." E2P2M 2:26*

Category N° 7: In the self-management process, the participation of the healthcare team and system is imperative. The healthcare team, composed of an interdisciplinary group of professionals, plays a relevant role in supporting the process with knowledge and education. Their contributions, through informational, educational, and coaching support, become facilitating factors for people with colostomies due to colorectal cancer. 

Within the Colombian healthcare system, it is imperative for patients to know and learn to navigate the administrative procedures required to access health services, such as authorizations and other processes. It is a relevant process due to the ongoing need for access to healthcare services, medical orders, and the timely provision of essential supplies or medications required for symptom management.

*"Well, they gave me the prescription to pick them up at the institutional pharmacy, and I went there with it, with the prescription. And there they simply handed them over—I didn't know the brand or anything. They always gave me that, Convatec." E2P2M 2:67*

Category N° 8: The religious influence is greatly recognized by individuals living with a colostomy, as the condition brings about drastic life changes. Spiritual support, whether through a relationship with God, prayer, or religious practices, is perceived as a source of strength and encouragement to overcome situations, becoming a prioritized aspect of their lives.

*"Yes, because I feel like God told me, 'You keep doing the same thing, so I'm going to put a stop to it—to make you wake up.' I don’t know, maybe He's putting this in my path because of something I did wrong, you know what I mean?" E8P8M 8:23*

## Discussion

The findings of this research regarding the difficulties associated with living with a colostomy are consistent with previous research on the physical effects of ostomies and their impact on physical well-being. Common issues include odor, gas, bowel elimination, sleep disturbances, leakage, colostomy care, and colostomy-related problems^16^,^18^,^27^. However, it is important to highlight specifically the difficulties in accessing essential colostomy care supplies. 

Living a new reality is described as an opportunity for reflection about experiences within a supportive environment, fostering acceptance of the condition and improved control of physical symptoms^16^,^28^. However, most of the characteristics, such as difficulty in adaptation, helping others, and difficulties in adjusting to change, are relevant aspects of this research. 

Regarding receiving social support, this study observed some contrasts with previous research related to the roles of family and social support. Research mentions maintaining marital relationships, performing family and social roles, the appreciation of familial care, and the need for support beyond the biological family, highlighting that social facilitation includes social influence, social support, and negotiated collaboration^16^,^22^,^29^. In addition, the support of other sources of information, such as the internet, has already been highlighted^17^. 

Knowledge about colostomy care and the ability to resolve doubts become relevant. Receiving information, education, and training enhances individuals' ability to manage the consequences of cancer and its treatment^28^. Notably, this study adds that personalizing the relationship with the colostomy during the adaptation process, such as nicknaming it, can foster a sense of confidence and ownership. Managing emotions and feelings was another common component. Participants found that this practice helps reframe their illness experience and puts things in perspective. This helped them cope better with challenges and manage distress more effectively^28^. 

The social effects of having a colostomy, such as difficulties attending meetings, traveling, and using transportation, along with financial burden, are consistent with prior data obtained on recreation and social engagement, followed by performing activities of daily living, and the added financial and emotional strain on families^18^,^30^. 

In terms of colostomy self-management skills, participants' use of homemade items for managing their colostomy care is particularly noteworthy. However, self-management strategies are related to the complexity of the disease, care, treatment data, and experimentation with new methods^16^,^17^. Moreover, detailed dietary practices were emphasized as a fundamental self-management strategy, as diet can reduce the risk of recurrence and prevent uncomfortable moments^29^. 

Regarding self-management with the healthcare team and health system, the presence of facilitators, such as emotional and informational support, was identified as important by the participants in self-management interventions. Accessibility of healthcare providers may serve as either a risk or protective factor, given that effective self-management relies on the collaboration with the care team^22^,^28^. However, this study points out that patients’ relationship with the health system depends on the structure of their insurance or healthcare enrollment and the contextual functioning of the health system, which may vary by country. 

The spiritual aspect, the search for God, and God’s empowerment were very significant components of people's lives in general. This feature provides support for difficult experiences and is a tool for getting help, support, and strength to continue a process, such as a colostomy due to cancer^16^,^29^. 

The study was not without its limitations. Data collection and transcription took longer than anticipated, and other challenges included limited privacy during some interviews and occasional audio recording issues. These factors were acknowledged and addressed to ensure they did not compromise the achievement of the study’s objectives. 

## Conclusions

Experience is a process involving individual situations. However, there are situations experienced by most participants that include difficulties related to odors, leakages, and gas, among others, dietary management and control, self-management practices, and ostomy care. Factors influencing self-management in adults with colostomy due to colorectal cancer include the strength of their spiritual dimension, the presence of social support, coping with colostomy difficulties, and experiencing psychosocial changes. 

Self-management behaviors and practices in adults with colostomy due to colorectal cancer that influence their experience include practicing colostomy self-management skills, living in a new reality, adapting to the colostomy, and learning self-management in collaboration with the healthcare team and system. This new knowledge contributes to the development of interventions that can have a better impact on the quality of life and experience of individuals with colostomies due to colorectal cancer. 
